# Validation of the Norma Latina Neuropsychological Assessment Battery in Patients with Alzheimer’s Disease in Mexico

**DOI:** 10.3390/ijerph191811322

**Published:** 2022-09-08

**Authors:** Silvia Núñez-Fernández, Diego Rivera, Eva María Arroyo-Anlló, Xóchitl Angélica Ortiz Jiménez, Borja Camino-Pontes, Ricardo Salinas Martínez, Juan Carlos Arango-Lasprilla

**Affiliations:** 1 Biocruces Bizkaia Health Research Institute, 48903 Barakaldo, Spain; 2 Neuroscience Institute of Castilla-León, University of Salamanca, 37007 Salamanca, Spain; 3 Department of Health Sciences, Public University of Navarre, 31006 Pamplona, Spain; 4 Instituto de Investigación Sanitaria de Navarra (IdiSNA), 31008 Pamplona, Spain; 5 Department of Psychobiology, Neuroscience Institute of Castilla-León, University of Salamanca, 37007 Salamanca, Spain; 6 Faculty of Psychology, Autonomous University of Nuevo León, Monterrey 64460, Mexico; 7 Biomedical Research Doctorate Program, University of the Basque Country (UPV/EHU), 48940 Leioa, Spain; 8 Department of Geriatrics, “José Eleuterio González” University Hospital, Autonomous University of Nuevo León, Monterrey 64460, Mexico; 9 Department of Psychology, Virginia Commonwealth University Richmond, Richmond, VA 23284, USA

**Keywords:** Alzheimer’s disease, neuropsychological assessment, cognitive problems, Latin America, Mexico

## Abstract

To our knowledge, this is the first study reported in the literature that has validated the Norma Latina Battery in a population of people with Alzheimer’s disease (AD) in Mexico. The objective of the study was to determine the discriminant validity of the Norma Latina Battery in a group of Mexican individuals with AD and a group of heathy controls (HC). The Norma Latina Battery was administered to 234 Mexican participants (117 HC and 117 individuals with AD). Results show that: (1) the Norma Latina Battery has high discriminative capacity between groups in all domains; (2) participants with AD presented worse scores in each of the cognitive domains compared to the HC and a greater number of low scores in each of the established thresholds or cut-off points; and finally, (3) the Norma Latina Battery had optimal sensitivity and specificity, especially when a set was observed ≥5 scores below the 10th percentile or ≥4 scores below the 5th percentile. In conclusion, it is recommended that both clinicians and researchers use this battery in the evaluation of Mexican people with AD to better understand the prognosis of the disease and its subsequent treatment.

## 1. Introduction

Alzheimer’s disease (AD) is a progressive and disabling neurodegenerative disease [1,2,3,4,5]. According to the WHO, it is estimated that more than 55 million people in the world currently live with dementia, and that by 2050 this number will reach 139 million [2]. This increase will be especially dramatic in underdeveloped or middle- or low-income countries such as in Latin America [3,6].

In Latin America, the prevalence of dementia is 7.1%, with AD being the most frequent type (56.3%) [7,8]. It is estimated that by 2040, there will be around 9.1 million people with AD in Latin America [4], which will generate high costs at personal and family levels to be able to provide the necessary care [9,10].

As AD is a serious and irreversible disease, it is usually characterized by the presence of physical [5], emotional and behavioral [5,11], and cognitive problems that interfere with the performance of activities of daily living (ADLs) [12,13]. At the cognitive level, AD is characterized by the presence of alterations in memory [5,14], processing speed [15], executive functions and attention [5,11,16], language [5,11], orientation, and abilities visuospatial [5,11,16].

The evaluation and diagnosis of these cognitive alterations is essential to document the progression of the disease and plan for its subsequent treatment [12,17,18]. Currently, there are several instruments and batteries widely used internationally with the aim of understanding the neuropsychological profile of this disease. These include: Consortium to Establish a Registry for Alzheimer’s Disease–CERAD [19,20,21]; Repeatable Battery for the Assessment of Neuropsychological Status–RBANS-3 [22]; Wisconsin Registry for Alzheimer’s Prevention [23]; Dementia Rating Scale-2–DRS-2 [24]; and Examination for Mental Disorders of Older People with Down’s Syndrome and Others with Intellectual Disabilities–CAMDEX-DS [25], among others.

The majority of these instruments have been standardized and validated in different countries around the world such as Australia [26], USA [27,28,29,30], Greece [31], Russia [32], Korea [33], Turkey [34], France [35], Spain [36,37], and Colombia [38,39,40], among others. In general, most of these tests have been developed in Anglo-Saxon countries, and the few that have been adapted and validated in Spanish have mostly been carried out in Spain [41]. There are currently very few standardization and validation studies of these neuropsychological batteries in the population with AD in Latin America. For example, Porto et al. [42] conducted a study to verify the diagnostic accuracy of the Brazilian version of the DRS in the diagnosis of patients with mild dementia in AD. This study found that the DRS showed good accuracy in discriminating patients with mild AD and controls [42]. On the other study, Aguirre-Acevedo et al. [38] in Colombia conducted a study with the aim of establishing the validity and reliability of the CERAD-Col and found that it was valid and reliable for the diagnosis of AD in a Spanish-speaking population over 50 years old. Finally, in Chile, Grandi et al. conducted a study with the objectives of: (1) adapting The Frontal Assessment Battery (FAB); (2) analyzing its psychometric properties; (3) evaluating the sociodemographic influence on FAB performance in a sample of healthy controls; and (4) developing normative data in healthy population. This study found that the FAB is a useful tool to discriminate between healthy people and people with dementia [43]. It is important to have accurate and valid population-specific tests because biological [44] and cultural [45] differences have been documented across populations. Despite the research noted above, very few studies in Spanish-speaking countries have developed or validated batteries for people with AD; and those that exist have a series of limitations, such as: (1) they were validated in most cases in small samples; (2) in some studies the dementia group was not only made up of people with AD, but of people with mild cognitive impairment and different types of dementia; and (3) they did not take into account illiterate individuals in the sample of patients with AD, despite the large number of patients who currently exist with these characteristics who have dementia.

The diagnosis of AD fundamentally requires a reliable neuropsychological evaluation, but the great scarcity of adapted, validated, and standardized evaluation tools in the Latin American population belies a lack of normative data in these countries, one of the greatest existing limitations for professionals in these countries to adequately evaluate and diagnose their patients [40,46,47,48]. These limitations increase the probability of inappropriate diagnoses when these types of tools are used [49], which is troublesome because the population with dementia is one of the clinical populations that most frequently undergoes neuropsychological consultation [46].

Recently, a multicenter study called Norma Latina has been carried out in which a group of ten neuropsychological tests have been scaled in healthy people from 12 Latin American countries, Spain, and Portugal [41,50,51,52,53,54]. This study has ushered a dramatic advance in this area, since the generation of these normative data makes it possible to investigate the usefulness of this battery in clinical populations such as AD. Therefore, the objectives of this study are: (1) to determine the discriminant validity of the Norma Latina Battery in a group of Mexican people with AD and a control group of Mexican people; (2) to describe the neuropsychological profile of the group of people with AD through the use of the Latin Standard Battery; and (3) to determine the sensitivity and specificity of the Latin Standard Battery between both groups to be able to establish a cut-off point. It is hoped that the results of this study will contribute to the accessibility of validated tools that allow neuropsychologists to assess the cognitive status of Spanish-speaking people with AD residing in Mexico, as well as being able to monitor the progression of the disease and the effects of treatments.

## 2. Materials and Methods

### 2.1. Participants

The sample consisted of 234 Mexican participants divided into two groups. The first group was made up of 117 Mexican adults diagnosed with AD, with a mean age of 77.7 (SD = 6.3) years of age and mean schooling of 6.7 (SD = 4.5) years. The second group of participants was composed of 117 healthy adults from the Norma Latina database for Mexico [41], matched by age (U = 6433.5; *p* = 0.427; r = 0.056), education (U = 6407.5; *p* = 0.395; r = 0.052) and gender (*X*^2^ = 0.00; *p* = 1.00; r = 0.00). For more information see Table 1.

The inclusion and exclusion criteria for participants with AD were: (a) being Mexican and residing in Mexico at the time of the evaluation; (b) being between 50 and 90 years old at the time of the evaluation; (c) having a clinical diagnosis of AD, according to the diagnostic criteria of the National Institute of Neurological and Communicative Diseases and Stroke/Alzheimer’s Disease and Related Disorders Association (NINCDS-ADRDA) [55]; (d) speaking Spanish as their native language; (e) knowing how to read and write at the time of the evaluation; (f) not having a premorbid history of central nervous system diseases such as, stroke, epilepsy, multiple sclerosis, brain tumor or traumatic brain injury, among others; (g) being free from active or uncontrolled systemic diseases associated with cognitive impairment, such as diabetes mellitus, hypothyroidism, and vitamin B12 deficiency; (h) not having severe behavioral deficits; (i) not having severe sensory deficits, such as vision and/or hearing loss that could negatively affect the administration of the tests and/or the performance of the participants; (j) not having a premorbid history of psychiatric problems such as major depression, bipolar mood disorder, and psychosis; (k) not having a premorbid history of neurological problems; (l) not having a prior history of mental retardation; (m) not having a previous history of learning problems; (n) not having a history of abuse of alcohol or other psychotropic substances; (o) not consuming medications unsupervised by a medical professional, and (p) not having a history of consuming medications for chronic pain.

The inclusion and exclusion criteria for healthy participants were: (a) being Mexican and residing in Mexico at the time of the evaluation; (b) being between 50 and 90 years of age at the time of the evaluation; (c) knowing how to read and write; (d) having Spanish as their native language; (e) having completed at least one year of formal education; (f) having a score ≥23 on the MMSE [56,57]; (g) having a score ≥90 on the Barthel Index [58]; (h) having a score ≤4 in the Patient Health Questionnaire-9 (Patient Health Questionnaire-9–PHQ-9) [59]; (i) not having a premorbid history of central nervous system diseases such as, stroke, epilepsy, multiple sclerosis, brain tumor or traumatic brain injury, among others; (j) being free from active or uncontrolled systemic diseases associated with cognitive impairment, such as diabetes mellitus, hypothyroidism and vitamin B12 deficiency; (k) not having a premorbid history of psychiatric illness such as major depression, bipolar illness and psychosis, among others; (l) not having severe sensory deficits, such as vision and/or hearing loss that could negatively affect the administration of the tests and/or the performance of the participants; (m) not consuming psychiatric or other drugs that may affect the person’s cognitive performance; (n) not taking medications for chronic pain, such as monoamine oxidase inhibitors–MAOIs; and (o) not abusing alcohol or other psychotropic substances before or during the time of the evaluation. For more information on the exclusion and inclusion criteria see Guàrdia-Olmos et al. [41].

### 2.2. Instruments

The neuropsychological battery was composed of nine neuropsychological tests most used by clinicians and researchers in Latin American countries [46]: Rey-Osterrieth Complex Figure (ROCF) [60,61,62]; Hopkins Verbal Learning Test-Revised (HVLT-R) [63,64]; Modified Wisconsin Card Sorting Test (M-WCST) [65,66]; Stroop Color-Word Interference Test (Stroop test) [39,67,68]; Verbal Fluency Test (VFT-Phonological and semantic) [69,70,71,72,73]; Boston Naming Test (BNT) [74,75,76]; Symbol Digit Modalities Test (SDMT) [77,78,79,80,81]; Trail Making Test (TMT) [82,83,84,85]; and Brief Test of Attention (BTA) [86]. For more details by each test, please see Supplementary Material S1.

### 2.3. Procedure

The Research and Ethics Committee of the Hospital Universitario Dr. José Eleuterio González [Number GE16-00001] (Monterrey, Mexico) approved this study, and it was conducted in accordance with the Declaration of Helsinki. The participants with AD were patients who attended medical follow up at the Hospital Universitario Dr. Jose Eleuterio Gonzalez. All patients were evaluated by an interdisciplinary Geriatric team and were screened for cognitive, affective, and physical dysfunction. AD diagnosis was supported with medical history, neuropsychology testing, metabolic tests, and magnetic resonance. A multidimensional treatment was then initiated. For this study, those participants who met the inclusion/exclusion criteria completed an approximately 2-h neuropsychological evaluation. Data collection from AD participants began in 2017 and ended in 2019. Data from healthy participants were extracted from the Norma Latina database for Mexico, whose collection began in 2013 and ended in 2014 [41]. All participants in both groups were volunteers and received no financial compensation for their participation. For more information on the study procedure in healthy participants, see Guàrdia-Olmos et al. [41].

### 2.4. Data Analyses

The Kolmogorov–Smirnov and Levene’s tests were used to evaluate normal distribution and homoscedasticity in the quantitative variables in both groups; most of the scores did not meet the assumptions of normality and homoscedasticity (for more details please see Supplemental Material S2), so the Mann–Whitney U test was used to compare the direct scores between both samples (participants with AD and healthy participants). Each direct score obtained from the Norma Latina Battery was transformed to both a z-score (z_i_) and a percentile using published normative data for HVLT-R [87,88], M-WCST [89], SDMT [90], TMT [91], BNT [92], BTA [93], Verbal Fluency Test (Semantic Verbal Fluency Test and Phonological Verbal Fluency Test) [69,94], ROCF [95], and Stroop test [96] in the Mexican population. Percentiles were used to estimate the rate of low scores for each participant at various cutoff points: (a) below the 25th percentile; (b) below the 16th percentile; (c) below the 10th percentile; (d) below the 5th percentile; and (e) below the 2nd percentile. The Mann–Whitney U test was used to assess the difference between the number of low scores for healthy participants and participants with AD from 24 z-scores (z_i_) obtained from the tests; four cognitive domains were created (composite scores; Z_c_): Executive Function [5 scores (M-WCST Categories, M-WCST Perseveration errors, M-WCST Total errors, Stroop Word-Color and Stroop Interference)]; Attention and Processing Speed [6 scores (SDMT, TMT-A, TMT-B, Stroop-Word, Stroop-Color and BTA)]; Language [8 scores (Letters F, A, S, M, Animals, Fruits, Occupations and BNT)]; and Learning and Memory [5 scores (ROCF copy, ROCF Recall, HVLT-R Total learning, HVLT-R Delay ed recall and HVLT-R Recognition)]. The domains were created following the classification suggested by various authors [88,97,98]. Each composite score (Z_c_) was created using Stouffer’s Z method [99]. The Mann–Whitney U test was used to compare groups according to these four domains. For all comparisons, effect size (r) was calculated to determine significant differences between groups. Effect sizes were interpreted as small when r ≥ 0.1, medium when r ≥ 0.3, and large or moderate when r ≥ 0.5.

Finally, Receiver Operating characteristic (ROC) analysis was carried out to determine whether the neuropsychological battery discriminated between healthy participants and participants with AD using the rate of low scores and performance in each proposed cognitive domain. The curve is created by plotting the true positive rate (TPR; also known as sensitivity) against the false positive rate (FPR; also known as specificity) for various cut-off points [100]. The Area Under the Curve (AUC) was calculated as a measure of accuracy or precision, where high precision was considered at a value equal to 0.9 or higher, moderate precision at a value between 0.7 and 0.9, and low precision at a value between 0.5. and 0.7 [101]. In addition, the Youden index [102] and the Union index [103] were calculated to determine the optimal cut-off point for the number of low scores below the 10th percentile and below the 5th percentile to discriminate between healthy participants and AD participants. The data were analyzed with SPSS version 26 for Mac [104].

## 3. Results

The Mann–Whitney U tests showed that there were significant differences between both groups (AD and healthy) in all direct scores of the neuropsychological tests except for M-WCST Total Error scores (*p* = 0.172, r = 0.089) (see Table 2). Furthermore, more than half (58.3%) of the effect sizes of the various neuropsychological test scores were large.

Normative data from the Mexican population were used to convert each direct score to z_i_ and percentiles adjusted for the demographic characteristics of each participant and to determine the base rate of low scores. The Mann–Whitney U tests showed significant differences between the distributions of low scores between the AD and healthy groups (*p*’s < 0.001; see Table 3), where the AD group showed a greater number of low scores in each of the established cut-off points (25th, 16th, 10th, 5th, and 2nd percentiles). For example, at the <25th percentile cutoff point, the AD group had a median of 17 low scores versus the healthy group, which had a median of 6 low scores; and in the <10 percentile borderline (whose threshold is commonly used to reflect poor and/or borderline patient performance) the AD group had a median of 12 low scores, while the healthy group had a median of two low scores. Furthermore, all comparisons showed large effect sizes (r’s ≥ 0.627).

AUCs indicated a high degree of accuracy in discriminating between AD and healthy participants at each of the cut-off points (see Table 3). The cut-off points with maximum Youden Index (J) and minimum Union Index (UI) showed that the optimal cut-off point is ≥5 (Sensitivity = 0.921 and Specificity = 0.850) for the number of low scores in the battery below the 10th percentile. Regarding the number of scores low below the 5th percentile, the potential cutoff point was ≥4 (Sensitivity = 0.841 and Specificity = 0.894; see Table 4).

**Figure 1:**
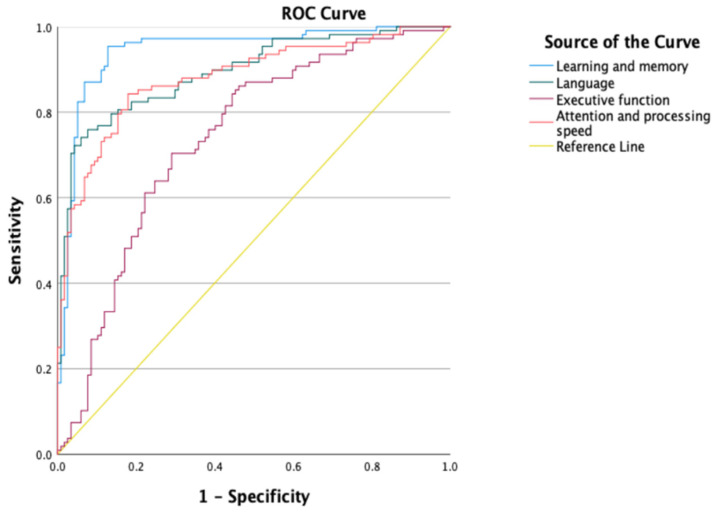
ROC curves representing different cognitive domains.

Significant differences were observed between both groups in all cognitive domains where AD participants presented worse median Z-scores in each cognitive domain compared to healthy participants (see Table 5). The AUCs for each cognitive domain showed a moderate–high degree of accuracy in discriminating between AD and healthy participants in each cognitive domain (Executive Function [AUC = 0.738, CI = 0.672, 0.803]; Attention and Processing Speed [AUC = 0.880, CI = 0.834, 0.925]; Language [AUC = 0.895, CI= 0.853, 0.936]; and Learning and Memory [AUC = 0.944, CI = 0.911, 0.976] (see Figure 1).

## 4. Discussion

To our knowledge, this is the first study reported in the literature that has validated the Norma Latina Battery in a population of people with AD in Mexico. The objectives of the study were to determine the discriminant validity of the Norma Latina Battery in a group of Mexican people with AD and a control group of Mexican people to describe the neuropsychological profile of the group of individuals with AD, as well as to determine the sensitivity and specificity of the battery between both groups and to be able to establish a cut-off point. The results showed that: (1) the Norma Latina Battery has high discriminative capacity between participants with AD and healthy participants in all domains, mainly in the domains of learning and memory and language domains; (2) participants with AD presented worse scores in each of the cognitive domains compared to the control group and a greater number of low scores in each of the established thresholds or cut-off points (25th, 16th, 10th, 5th and 2nd percentiles); and finally, (3) the Norma Latina Battery had optimal sensitivity and specificity, specifically when a set was observed ≥5 scores below the 10th percentile or ≥4 scores below the 5th percentile.

Currently, there are different studies that have been carried out with the purpose of validating and assessing different screening tests for people with dementia in the Spanish-speaking population [57,105,106]. However, very few studies to date have been carried out with the aim of validating neuropsychological assessment batteries in individuals with Alzheimer’s disease in Latin America [38,42,43,107,108].

The results of this study concur with those reported in previous studies in which neuropsychological test batteries are useful tools to discriminate between healthy people and people with neurodegenerative diseases [38,42,43,109,110]. Specifically, in this study the group of participants with AD presented significantly lower scores compared to the healthy group, a fact widely reported also by other studies [38,42,43,108]. In addition, the AUCs were moderate and large (AUC’s ≥ 0.78) when evaluating the degree of discrimination between the group of healthy participants and the AD group, aspects that have also been previously reported in the literature [42,108].

On the other hand, it is important to highlight that there are several methodological differences between the present study and previous research. For example: (1) Very few studies have been conducted with the aim of validating neuropsychological test batteries in people with dementia in Latin America [38,42,43,107,108]. Some of those that have been done combine patients with different types of dementia or have been done with Portuguese-speaking people [42,107]; (2) The total number of tests and scores is different between the studies, where in this study Norma Latina consists of nine tests and 24 test-scores, while in similar studies batteries made up of eight [38], seven (INECO Frontal Screening [IFS]) [108], or six (Frontal Assessment Battery [FAB]) [43,108] test-scores, except for the study by Porto et al. [42] who studied 144 test-scores; (3) The sample size of the groups is different, especially in the AD group, where the present study has a sample of 117 participants with AD, while Custodio et al. [108] analyzed 35 participants and Fonseca et al. [107] 11, while Aguirre-Acevedo et al. [38] and Grandi et al. [43] studied 151 and 150 participants with AD, respectively; (4) The number of cognitive domains that the batteries evaluate vary, for example [43,108] present validation of IFS and FAB, these batteries specialize in measuring executive function, while Norma Latina Battery groups the 24 test-scores in four cognitive domains commonly used in the clinic (Executive Function, Attention and Processing Speed, Language and Learning and Memory) in a similar way as Porto et al. [42] that groups by domains of Attention, Initiation/Perseveration, Construction, Conceptualization, and Memory; (5) In the present study, the estimation of the most used cut-off points in the clinic (25th, 16th, 10th, 5th, and 2nd) was carried out from a multivariate approach [98], which allowed adjusting each test-score to the demographic variables of the participants through the Multivariate base rates of low scores. However, studies such as those conducted by Custodio et al. [108], Grandi et al. [43], Porto et al. [42] and Fonseca et al. [107] did not make any type of adjustment, since they assumed that because there were no significant differences between the sociodemographic characteristics of the patients, the samples were paired, so eliminating this effect; (6) Another notable difference is the different statistics used to determine the optimal cut-off point from the ROC curve. While objective tools such as the Youden index and Index of Union were used in the present study, in the studies by Custodio et al. [108], Grandi et al. [43], and Porto et al. [42], no techniques were used to select the cut-off point; Finally, (7) in the present study the effect size was estimated for each of the comparisons made, indicating the magnitude of the differences regardless of statistical significance [111] while in the other studies it was not done.

In summary, the findings of the present study confirm the usefulness of a battery of neuropsychological tests for the discrimination of healthy people and people with dementia as previously reported in the literature. However, the importance of this study lies in the fact that the battery of tests used was in Spanish, and it was carried out with Mexican patients with AD. This battery consisted of tests that evaluated the main cognitive domains that are usually altered in people with AD at clinical level. Moreover, the statistical analysis allowed us to know the discriminant capacity of the battery by domain, and the optimal cut-off points that will allow the clinician to have a valid screening tool in the Mexican population. Additionally, the results of this study provide validity to the previous methodology used for generating normative data in this Mexican population. This is relevant, because test performance is influenced by many factors such as sociodemographic (e.g., age, education, sex) and cultural characteristics affecting the adequate interpretation of test scores [112]. These cultural effects are observed within Latin American countries, observing differences in the patterns of scores between countries [49,50].

### 4.1. Implications

To date, there are no validated neuropsychological batteries in Mexico for individuals with AD. Therefore, the results of this study are of the utmost importance since they allow clinicians who work daily with people with AD to apply a useful tool in identifying the cognitive deficits of patients in the region. In addition, this battery will provide valid information for the evaluation of the strengths and weaknesses of this patient population, which will help to better understand the prognosis of the disease and help its subsequent treatment.

### 4.2. Limitations

The results of this study should be interpreted in light of the following limitations: (1) this study was conducted with patients with AD in Mexico, so the results cannot be generalized to other Latin American countries; (2) the study was carried out only with people with AD, so the ability of the Norma Latina Battery to discriminate healthy people from people with other types of dementia or people with mild cognitive impairment is unknown. Future studies should examine the validity of this battery in other populations with dementia; (3) AD is a neurodegenerative disorder, so as the disease progress, the Norma Latina Battery discrimination capacity may vary; and (4) the Norma Latina Battery was composed of tests that evaluated attention, language, executive functions, memory, and learning, but this battery should not be used to evaluate other cognitive domains such as motor function, visuospatial and visuo-constructive abilities in individuals with AD.

## 5. Conclusions

AD requires a comprehensive care approach, starting from an adequate assessment. The Norma Latina Battery successfully discriminated between individuals with AD and healthy controls. For this reason, it is recommended that both clinicians and researchers use this battery in the evaluation of Mexican people with AD. In addition, the Norma Latina Battery can also be a useful tool at the rehabilitation stage as it may be used to know how effective an intervention can be for people with AD. Future studies should investigate the usefulness of this tool in discriminating other clinical populations such as people with other neurodegenerative diseases, people with strokes, head injuries, and epilepsy, among others. Likewise, studies like this one should be carried out in other Latin American countries.

## Figures and Tables

**Table 1:** Demographic Characteristics of the Sample.

		HC (*n* = 117)			AD (*n* = 117)			Statistic	*Sig*.	Effect Size (*r*)
		Median	Min.	Max.	Median	Min.	Max.			
Age		78	57	88	78	57	88	U = 6433.5	0.427	0.056
Education		6	1	20	6	0	20	U = 6407.5	0.395	0.052
Sex	Female		91	77.8%		91	77.8%	*X*^2^ = 0.00	1.00	0.000
	Male		26	22.2%		26	22.2%			

Note: HC = Healthy Control; AD = Alzheimer’s disease; Min = Minimum; Max = Maximum.

**Table 2:** Comparison between groups on neuropsychological test scores.

Test-Score	Group	Median	Min.	Max.	Mann–Whitney U	*Sig*.	*r*
ROCF Copy	AD	7.0	0.0	36.0	1973.000	<0.001	0.609 ^†††^
	HC	30.0	9.5	36.0			
ROCF Recall	AD	0.0	0.0	20.0	889.000	<0.001	0.759 ^†††^
	HC	13.5	0.0	34.0			
Stroop Word	AD	50.5	7.0	100.0	3219.500	<0.001	0.341 ^††^
	HC	70.0	21.0	110.0			
Stroop Color	AD	31.0	0.0	87.0	2609.500	<0.001	0.415 ^††^
	HC	49.0	2.0	93.0			
Stroop Word—Color	AD	8.0	0.0	40.0	1192.000	<0.001	0.630 ^†††^
	HC	26.0	0.0	52.0			
Stroop Interference	AD	−7.5	−31.0	26.0	3001.000	<0.001	0.373 ^††^
	HC	−1.9	−16.0	17.9			
M-WCST Categories	AD	1.0	0.0	6.0	2948.000	<0.001	0.391 ^††^
	HC	3.0	0.0	6.0			
M-WCST Perseveration errors	AD	11.0	1.0	44.0	4025.500	0.001	0.226 ^†^
	HC	7.0	0.0	45.0			
M-WCST Total errors	AD	24.0	4.0	47.0	4951.500	0.172	0.089
	HC	18.0	0.0	47.0			
TMT-A	AD	100.0	35.0	100.0	2664.000	<0.001	0.438 ^††^
	HC	81.0	28.0	100.0			
TMT-B	AD	300.0	82.0	300.0	1167.500	<0.001	0.596 ^†††^
	HC	172.0	34.0	300.0			
BTA	AD	5.0	0.0	19.0	2856.500	<0.001	0.492 ^††^
	HC	12.0	0.0	20.0			
VFT Letter F	AD	4.0	0.0	14.0	2488.000	<0.001	0.547 ^†††^
	HC	9.0	1.0	23.0			
VFT Letter A	AD	4.0	0.0	15.0	2975.500	<0.001	0.486 ^††^
	HC	8.0	2.00	23.0			
VFT Letter S	AD	3.5	0.0	16.0	2843.000	<0.001	0.503 ^†††^
	HC	8.0	2.0	21.0			
VFT Letter M	AD	4.0	0.0	17.0	2834.500	<0.001	0.503 ^†††^
	HC	9.0	1.0	26.0			
VFT Animals	AD	7.0	0.0	21.0	1951.500	<0.001	0.619 ^†††^
	HC	14.0	5.0	24.0			
VFT Fruits	AD	6.0	0.0	19.0	2078.500	<0.001	0.603 ^†††^
	HC	12.0	4.0	21.0			
VFT Occupations	AD	4.0	0.0	14.0	1928.500	<0.001	0.623 ^†††^
	HC	9.0	4.0	20.0			
BNT	AD	24.0	0.0	53.0	1855.000	<0.001	0.631 ^†††^
	HC	45.0	0.0	60.0			
SDMT	AD	4.0	0.0	37.0	1411.500	<0.001	0.678 ^†††^
	HC	22.0	3.0	50.0			
HVLT-R Total learning	AD	9.0	0.0	22.0	1098.500	<0.001	0.726 ^†††^
	HC	17.0	6.0	29.0			
HVLT-R Delayed recall	AD	0.0	0.0	7.0	1235.500	<0.001	0.745 ^†††^
	HC	5.0	0.0	11.0			
HVLT-R Recognition	AD	8.0	0.0	12.0	3669.000	<0.001	0.406 ^††^
	HC	11.0	4.0	12.0			

*Note:* AD = Alzheimer’s disease; HC = Healthy Control; ROCF = Rey-Osterrieth Complex Figure; M-WCST = Modified Wisconsin Cart Sorting Test; TMT= Trail Making Test; BTA= Brief Test of Attention; VFT = Verbal Fluency Test; BNT= Boston Naming Test; SDMT = Symbol Digit Modalities Test; HVLT-R = Hopkins Verbal Learning Test–Revised; Min = Minimum; Max = Maximum; r = Effect size; ^†^ = Small effect; ^††^ = Medium effect; ^†††^ = Large effect.

**Table 3:** Comparison between groups on the number of test scores falling below specified percentile cutoffs.

Cut-Off	Group	Median	Min.	Max.	Mann–Whitney U	*Sig*.	*r*	AUC [CI_95%_]
<25th percentile	AD	17	7	24	457.000	<0.001	0.627 ^†††^	0.936 [0.901, 0.970]
	HC	6	0	23				
<16th percentile	AD	15	5	24	395.000	<0.001	0.640 ^†††^	0.945 [0.912, 0.977]
	HC	4	0	22				
<10th percentile	AD	12	2	23	391.500	<0.001	0.641 ^†††^	0.945 [0.912, 0.977]
	HC	2	0	19				
<5th percentile	AD	9	1	21	376.000	<0.001	0.649 ^†††^	0.947 [0.917, 0.978]
	HC	1	0	14				
<2nd percentile	AD	5	0	20	427.500	<0.001	0.653 ^†††^	0.940 [0.905, 0.975]
	HC	0	0	7				

Note: AD = Alzheimer’s disease; HC = Healthy Control; Min = Minimum; Max = Maximum; *r* = Effect size; ^†††^ = Large effect.

**Table 4:** Cut point and associated sensitivity and specificity values.

Cut Point	<5th Percentile				<10th Percentile			
	Se	Sp	*J*	UI	Se	Sp	*J*	UI
≥1	0.984	0.628	0.612	0.356	1.000	0.381	0.381	0.619
≥2	0.968	0.761	0.729	0.207	1.000	0.540	0.524	0.444
≥3	0.873	0.823	0.696	0.198	0.984	0.664	0.632	0.304
≥4	**0.841**	**0.894**	**0.735**	**0.159**	0.952	0.743	0.680	0.210
≥5	0.746	0.956	0.702	0.210	**0.921**	**0.850**	**0.771**	**0.119**
≥6	0.651	0.982	0.633	0.331	0.841	0.912	0.753	0.137
≥7	0.571	0.982	0.553	0.411	0.794	0.938	0.732	0.158

Note: Se = Sensitivity; Sp = Specificity; *J* = Youden index; UI = Index of Union; Lower bound and upper bound refer to the 95% confidence intervals of the AUC. Highlighted in bold the optimal point at cut-off point ≥4 at <5th percentile and at cut-off point ≥5 at <10th percentile.

**Table 5:** Average performance between groups by cognitive domain.

Domains	Group	Median (Z-Score)	SD	Mann–Whitney U	*Sig*.	*r*	AUC [CI_95%_]
Executive Function	AD	−2.03	1.55	3314.000	<0.001	0.403 ^††^	0.785 [0.726, 0.845]
	HC	−0.30	1.83				
Attention and Processing Speed	AD	−3.64	1.68	1551.000	<0.001	0.665 ^†††^	0.892 [0.849, 0.936]
	HC	−0.68	1.62				
Language	AD	−4.72	2.48	1334.000	<0.001	0.696 ^†††^	0.892 [0.849, 0.935]
	HC	−0.61	1.97				
Learning and Memory	AD	−4.18	2.36	721.000	<0.001	0.773 ^†††^	0.944 [0.912, 0.976]
	HC	0.25	1.69				

Note: AD = Alzheimer’s disease; HC = Healthy Control; SD = Standard Deviation; *r* = Effect size; ^††^ = Medium effect; ^†††^ = Large effect.

## Data Availability

Not applicable.

## Supplementary Materials

The following supporting information can be downloaded at: https://www.mdpi.com/article/10.3390/ijerph191811322/s1, Supplementary material S1: Brief Summary of each of the tests in the Norma Latina Battery and information on its reliability and validity; Table S1: Reliability for each cognitive domain; Supplementary material S2: Figure S1: Test-score distribution; Figure S2: Q-Q plot by each test-score; Table S2: Normality and homoscedasticity assumptions by each test-score. References [113,114] are cited in the Supplementary Materials.
