# Supplementary material for: Validation of the Norma Latina Neuropsychological Assessment Battery in Patients with Alzheimer’s Disease in Mexico
Source: Int J Environ Res Public Health. 2022 Sep 8;19(18):11322. doi: 10.3390/ijerph191811322 (PMC9517570; doi:10.3390/ijerph191811322)
Supplement: Supplementary file 1 [file ijerph-19-11322-s001.zip › Supplementary Material S1.pdf]

# Supplementary Material S1

*Brief description of each of the tests in the Norma Latina Battery and information on their reliability and validity.*

**Rey-Osterrieth Complex Figure (ROCF)**. The examiner administered the ROCF Figure A, which included the Copy portion, and Immediate Recall after a 3-min delay after the copy trial. The ROCF includes 18 elements, and the maximum score for each of the two tasks (Immediate and Delayed Recall) is 36. The Spanish-language ROCF manual scoring guidelines were followed [1]. This test has received psychometric support for both reliability and validity in past research for example with pediatric populations [2]. Reliability among pediatric populations tends to be high for both copy production ( $\alpha$ = 0.95) and recall production ($\alpha$ = 0.94) [2]. Others studies have showed that the reliability of this test is high, with a Cronbach's alpha of 0.828 for the copy, and 0.783 for the memory [3,4].

**Hopkins Verbal Learning Test-Revised (HVLT-R)**. In this study Form 5 was used. This form contains a list of 12 semantically related words in three categories (i.e., professions, sports, and vegetables). Three trials of successive learning are presented, in which the list of 12 words is read to the participant, and the correct answers of each learning trial are recorded. Total Recall is the sum of words recalled correctly in the three trials. After 20–25 min, the Delayed Recall and recognition phase occurs, where the subject is asked to recall all the words that they can remember from the initial list [5,6]. Some studies have estimated test-retest reliability in a sample of older adults and obtained coefficients within the accepted limits for the three learning tests (r's > 0.41) and I have also calculated the construct validity of the learning and retrieval measures [5].

**Modified Wisconsin Card Sorting Test (M-WCST)**. M-WCST consists of four stimulus cards and 48 response cards. Each card varies in shape (cross, circle, triangle, or star), color (red, blue, yellow, or green), and number (one to four). The objective is to correctly classify the stimulus card according to certain rule until completion of a category. The test continues until all six categories are classified or until the whole volume has been used [7]. Studies indicate that test-retest correlation coefficients showed significant values between the two applications for both total errors and total perseverations (r's > 0.34; p's < 0.01) [8].

**Stroop Color-Word Interference Test (Stroop test).** Stroop test consists of three pages, each with 100 components randomly organized into five columns. In the first page the participant must read aloud the words "Red," "Green," and "Blue" printed in black ink. In the second one, "color naming," the color (blue, green, or red) of each element "XXXX" must be named. And in the last one, "interference," the task is to name the color of the ink, inhibiting the reading of the word, which corresponds to the name of another color. The subject has 45 seconds to read aloud, as quickly as possible, the columns from left to right. Finally, the Interference Index was calculated with the formula: $WC – [(W \times C)/(W + C)]$, and indicates the degree to which the person has control over interference [9]. Some studies such as the one by Rodríguez- Barreto et al. found Pearson r correlations Stroop significant at 0.01 to -0.41 values for W; -0.35 for C; from -0.40 to -0.14 for WC and Interference. They were found a reliability higher than 0.70 in all Stroop scales [10]. Other studies using observational and longitudinal research evaluated reliability by test-retest in 848 participants from the general population, 151 people with Alzheimer's dementia and 36 people with Mild Cognitive Impairment and found that, in relation to speed as a cognitive function, a total internal consistency (95% confidence interval) with Cronbach's alpha values at W = 0.78, C = 0.75 and WC = 0.78. Regarding the general population, alpha values were found for W of 0.74, C of 0.72 and for WC of 0.74 [11].

**Verbal Fluency Test (VFT - Phonological and semantic).** In semantic VFT, the participants are required to produce (in 60 s) as many words as they can belong to a category (in this study, animals, fruits, and professions [12]. In terms of psychometric properties, correlational analyses analyzed the test–retest reliability within acceptable limits, with a Cronbach alpha of .74 [4,13]**.** On the other hand, several studies have observed correlations between this fluency test and other tests of this type (convergent validity), with moderate to high correlations being found in the case of the VFT - semantic (0.66 - 0.71) [14,15].

**Boston Naming Test (BNT)**. BNT requires examinees to name 60 pictures, which are presented in order of increasing difficulty. If the participant does not provide a correct answer spontaneously, the examiner provides a semantic clue (in case of misrecognition error) or phonological cue (when the semantic cue did not generate a response, or when the participant's erroneous spontaneous response did not reflect a misrecognition error). The Spanish version of the Kaplan et al. [16] was used for this study. In terms of psychometric properties, several general studies indicate that most of the test-retest reliability coefficients found are above 0.77 in different types of samples, for example, in patients with aphasia and older adults without brain damage [17,18].

**Symbol Digit Modalities Test (SDMT).** SDMT consists of converting symbols with shaped geometric figures in numbers according to a reference key at the top of the page. It must be performed in the order indicated, without skipping any element for 90 seconds [19]. Regarding the psychometric properties of this test, it was found that the reliability of the test has been demonstrated in various populations, including older adults (Cohen's d 0.263 to 0.351) [20]. On the other hand, other studies indicate that the test-retest correlations for SDMT, as well as the correlation between the different modes of test administration (written or oral), is 0.80 for the healthy population [21–23].

**Trail Making Test (TMT).** TMT consists of two trials: the Trail Making Test-Part A (TMT-A), whose goal the goal is to connect consecutively numbered semi-randomly distributed circles on a sheet of paper as quickly as possible by drawing lines between them, without lifting the pencil from the paper [24,25], and Trail Making Test-Part B (TMT-B) requires the subject to connect on a separate worksheet in ascending and alternating order the same number of circles which contain numbers and letters (i.e., 1-A-2-B-3, etc.) [26]. Test reliability has been demonstrated through inter-rater correlation coefficients that have been estimated at r = 0.99 for TMT- A and r = 0.93 in the case of TMT- B. In addition, construct validity has been demonstrated through factor analysis [27]**.**

**Brief Test of Attention (BTA)**. BTA consists of two parallel forms (Form N and L) which are presented orally to the subjects by the administrator. In Form N, 10 lists of letters and numbers (i.e., "M-6-3-R -2") are read out to the subject, the length of which will increase from 4 to 18 items. Subject indicates the number of numbers that have been read aloud to him/her, ignoring the letters presented. For the L form, the same process is followed, the subject must indicate the number of letters read aloud in each of the items, ignoring the numbers presented. The sum of the correct answers obtained in both forms is what constitutes the final BTA score and can vary from 0 to 20 [28]. Psychometric properties of the BTA obtained in the original validation conducted in the United States indicated good internal reliability with a coefficient alpha of 0.82 in a sample of healthy adults and children and 0.91 in a sample of healthy participants together with participants with various cognitive disorders [28]. Regarding the recurrent validity of the BTA, studies indicate that it correlates significantly with other measures of attention such as the

Digit Span Test (r = 0.53 backward and r = 0.43 forward), the Stroop Test (r = - 0.48 in TMT-A and r = - 0.55 in TMT-B) and the Stroop Test (r = 0.66 word, r = 0.68 color, r = 0.67 word-color) [28].

**Table S1.** Reliability for each cognitive domain.

| Cognitive domain | Numbers of test-scores | Cronbach's alpha |
|---|---|---|
| Executive Function | 4 scores | 0.682, $CI_{95\%}$ (0.603 - 0.749) |
| Attention & Processing Speed | 6 scores | 0.674, $CI_{95\%}$ (0.595 - 0.742 |
| Language | 8 scores | 0.871, $CI_{95\%}$ (0.844 -0.894) |
| Learning and Memory | 5 scores | 0.814, $CI_{95\%}$ (0.773 - 0.849) |

## References


1. Rey, A. *REY: Test de Copia y de Reproducción de Memoria de Figuras Geométricas Complejas.*; TEA Ediciones: Madrid, Spain, 2009.
2. Waber, D.P.; Holmes, J.M. Assessing Children's Copy Productions of the Rey-Osterrieth Complex Figure. *J. Clin. Exp. Neuropsychol.* **1985**, *7*, 264–280. doi:10.1080/01688638508401259.
3. Cortés, J.F.; Galindo, G.; Villa, M.; Salvador, J. La Figura Compleja de Rey: Propiedades Psicométricas. *Salud Mental* **1996**, *19*, 42–48.
4. Buré-Reyes, A.; Hidalgo-Ruzzante, N.; Vilar-López, R.; Gontier, J.; Sánchez, L.; Pérez-García, M.; Puente, A.E. Neuropsychological Test Performance of Spanish Speakers: Is Performance Different across Different Spanish-Speaking Subgroups? *J. Clin. Exp. Neuropsychol.* **2013**, *35*, 404–412. https://doi.org/10.1080/13803395.2013.778232.
5. Benedict, R.H.B.; Schretlen, D.; Groninger, L.; Brandt, J. Hopkins Verbal Learning Test—Revised: Normative Data and Analysis of Inter-Form and Test-Retest Reliability. *Clin. Neuropsychol.* **1998**, *12*, 43–55. https://doi.org/10.1076/clin.12.1.43.1726.
6. Brandt, J. The Hopkins Verbal Learning Test: Development of a New Memory Test with Six Equivalent Forms. *Clin. Neuropsychol.* **1991**, *5*, 125–142. https://doi.org/10.1080/13854049108403297.
7. Schretlen, D. *Modified Wisconsin Card Sorting Test Professional Manual.*; Psychological Assessment Resources Inc.: Odessa, FL, USA, 2010.
8. Bird, C.M.; Papadopoulou, K.; Ricciardelli, P.; Rossor, M.N.; Cipolotti, L. Monitoring Cognitive Changes: Psychometric Properties of Six Cognitive Tests. *Br. J. Clin. Psychol* **2004**, *43*, 197–210. https://doi.org/10.1348/014466504323088051.
9. Golden, C.J. *Manual Del Test de Colores y Palabras*; 5th ed.; TEA ediciones: Madrid, Spain, 2010.
10. Rodríguez-Barreto, L.C.; Pulido, N. del C.; Pineda-Roa, C.A. Propiedades psieométrieas del Stroop, test de colores y palabras en población colombiana no patológica. *Univ. Psychol.* **2016**, *15*, 255–272. https://doi.org/10.11144/Javeriana.upsy15-2.ppst.
11. Henao-Arboleda, E.; Muñoz, C.; Aguirre-Acevedo, D.C.; Lara, E.; Pineda, D.A.; Lopera, F. Datos Normativos de Pruebas Neuropsicológicas En Adultos Mayores En Una Población Colombiana. *Rev. Chil. Neuropsicol.* **2010**, *5*, 214–226.
12. Olabarrieta-Landa, L.; Rivera, D.; Galarza-del-Angel, J.; Garza, M.; Saracho, C.; Rodríguez, W.; Chávez-Oliveros, M.; Rábago, B.; Leibach, G.; Schebela, S.; et al. Verbal Fluency Tests: Normative Data for the Latin American Spanish Speaking Adult Population. *NeuroRehabilitation* **2015**, *37*, 515–561. https://doi.org/10.3233/./NRE-151279.
13. Tombaugh, T.N.; Kozak, J.; Rees, L. Normative Data Stratified by Age and Education for Two Measures of Verbal Fluency: FAS and Animal Naming. *Arch. Clin. Neuropsychol.* **1999**, *14*, 167–177.
14. Delis, D.C.; Kaplan, E.; Kramer, J.H. *Delis-Kaplan Executive Function System*; Psychological Corporation: San Antonio, TX, USA, 2001.
15. Riva, D.; Nichelli, F.; Devoti, M. Developmental Aspects of Verbal Fluency and Confrontation Naming in Children. *Brain Lang.* **2000**, *71*, 267–284. https://doi.org/10.1006/brln.1999.2166.
16. Kaplan, E.; Goodglass, H.; Barresi, B. *Evaluación de La Afasia y de Trastornos Relacionados*; 3rd ed.; Médica Panamericana: Madrid, Spain, 2005.
17. del Toro, C.M.; Bislick, L.P.; Comer, M.; Velozo, C.; Romero, S.; Gonzalez Rothi, L.J.; Kendall, D.L. Development of a Short Form of the Boston Naming Test for Individuals With Aphasia. *J. Speech Lang. Hear. Res.* **2011**, *54*, 1089–1100. https://doi.org/10.1044/1092-4388(2010/09-0119).

18. Flanagan, J.L.; Jackson, S.T. Test-Retest Reliability of Three Aphasia Tests: Performance of Non-Brain-Damaged Older Adults. *J. Commun. Disord.* **1997**, *30*, 33–43. https://doi.org/10.1016/S0021-9924(96)00039-1.
19. Smith, A. *Manual de Test. de Símbolos y Dígitos SDMT.*; Publicaciones de psicología aplicada.; TEA Ediciones: Madrid, Spain, 2002.
20. Wolinsky, F.D.; Vander Weg, M.W.; Howren, M.B.; Jones, M.P.; Dotson, M.M. A Randomized Controlled Trial of Cognitive Training Using a Visual Speed of Processing Intervention in Middle Aged and Older Adults. *PLoS ONE* **2013**, *8*, e61624. https://doi.org/10.1371/journal.pone.0061624.
21. Levine, A.J.; Miller, E.N.; Becker, J.T.; Selnes, O.A.; Cohen, B.A. Normative Data for Determining Significance of Test–Retest Differences on Eight Common Neuropsychological Instruments. *Clin. Neuropsychol.* **2004**, *18*, 373–384. https://doi.org/10.1080/1385404049052420.
22. Berrigan, L.I.; Fisk, J.D.; Walker, L.A.S.; Wojtowicz, M.; Rees, L.M.; Freedman, M.S.; Marrie, R.A. Reliability of Regression-Based Normative Data for the Oral Symbol Digit Modalities Test: An Evaluation of Demographic Influences, Construct Validity, and Impairment Classification Rates in Multiple Sclerosis Samples. *Clin. Neuropsychol.* **2014**, *28*, 281–299. https://doi.org/10.1080/13854046.2013.871337.
23. Lezak, M.D. *Neuropsychological Assessment*, 2nd ed.; Oxford University Press: New York, NY, USA, 1983.
24. Lezak, M.; Howieson, D.B.; Loring, D.W. *Neuropsychological Assessment*; 4th ed.; Oxford University Press: New York, NY, USA, 2004; ISBN 978-0-19-511121-7.
25. Reitan, R.M.; Wolfson, D. *The Halstead-Reitan Neuropsychological Test Battery: Theory and Clinical Interpretation*; Neuropsychology Press: Tucson, AZ, USA, 1985.
26. Drane, D.L.; Yuspeh, R.L.; Huthwaite, J.S.; Klingler, L.K. Demographic Characteristics and Normative Observations for Derived-Trail Making Test Indices. *Neuropsychiatry Neuropsycho. Behav. Neurol.* **2002**, *15*, 39–43.
27. Allen, D.N.; Thaler, N.S.; Barchard, K.A.; Vertinski, M.; Mayfield, J. Factor Structure of the Comprehensive Trail Making Test in Children and Adolescents with Brain Dysfunction. *Psychol. Assess.* **2012**, *24*, 964–972. https://doi.org/10.1037/a0028521.
28. Schretlen, D.; Bobholz, J.H.; Brandt, J. Development and Psychometric Properties of the Brief Test of Attention. *Clin. Neuropsychol.* **1996**, *10*, 80–89. https://doi.org/10.1080/13854049608406666.
