# Supplementary material for: Validation of the Norma Latina Neuropsychological Assessment Battery in Patients with Alzheimer’s Disease in Mexico
Source: Int J Environ Res Public Health. 2022 Sep 8;19(18):11322. doi: 10.3390/ijerph191811322 (PMC9517570; doi:10.3390/ijerph191811322)
Supplement: Supplementary file 1 [file ijerph-19-11322-s001.zip › Supplementary Material S2.pdf]

Supplementary Material S2

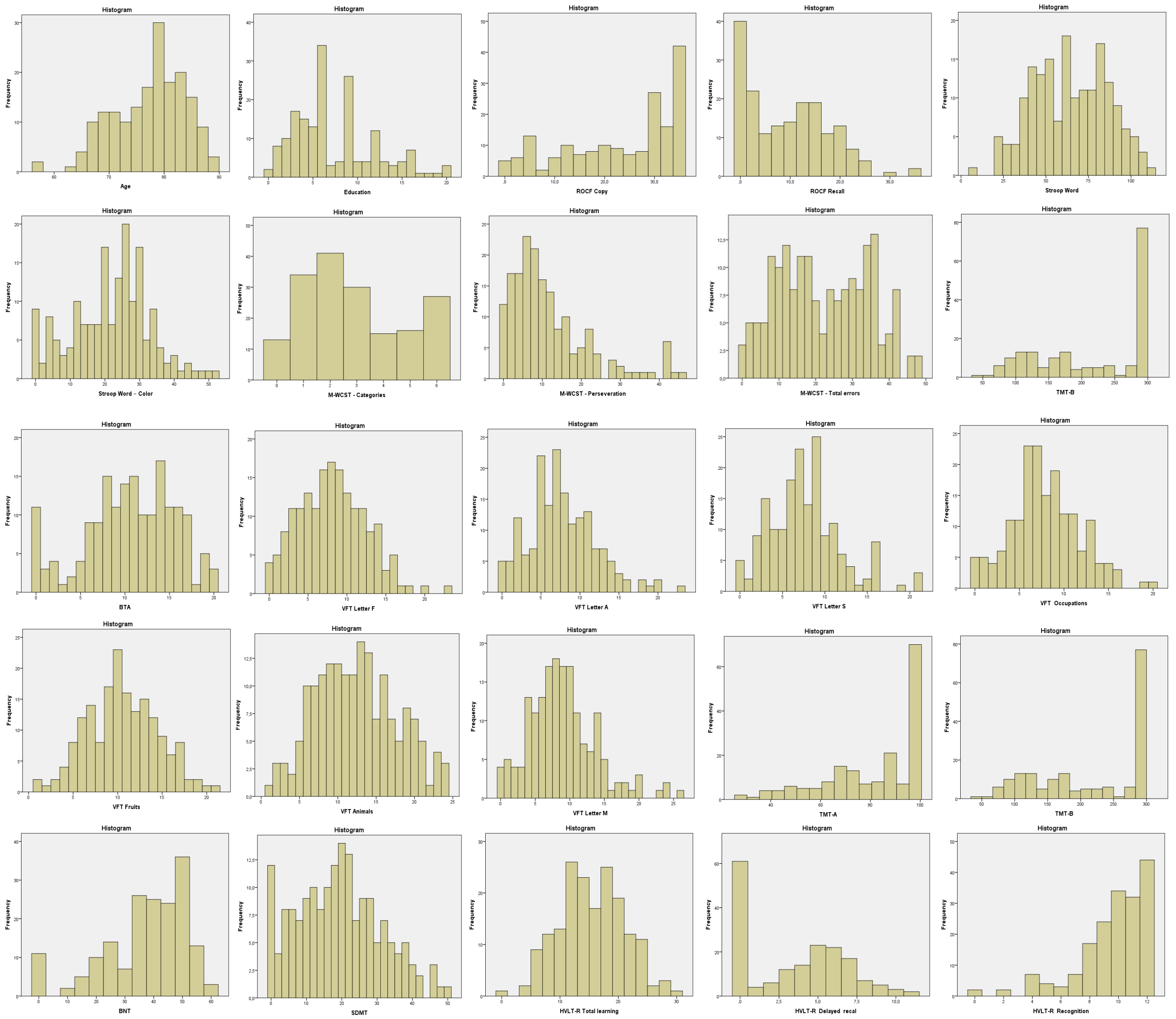

Figure S1. Test-score distribution.

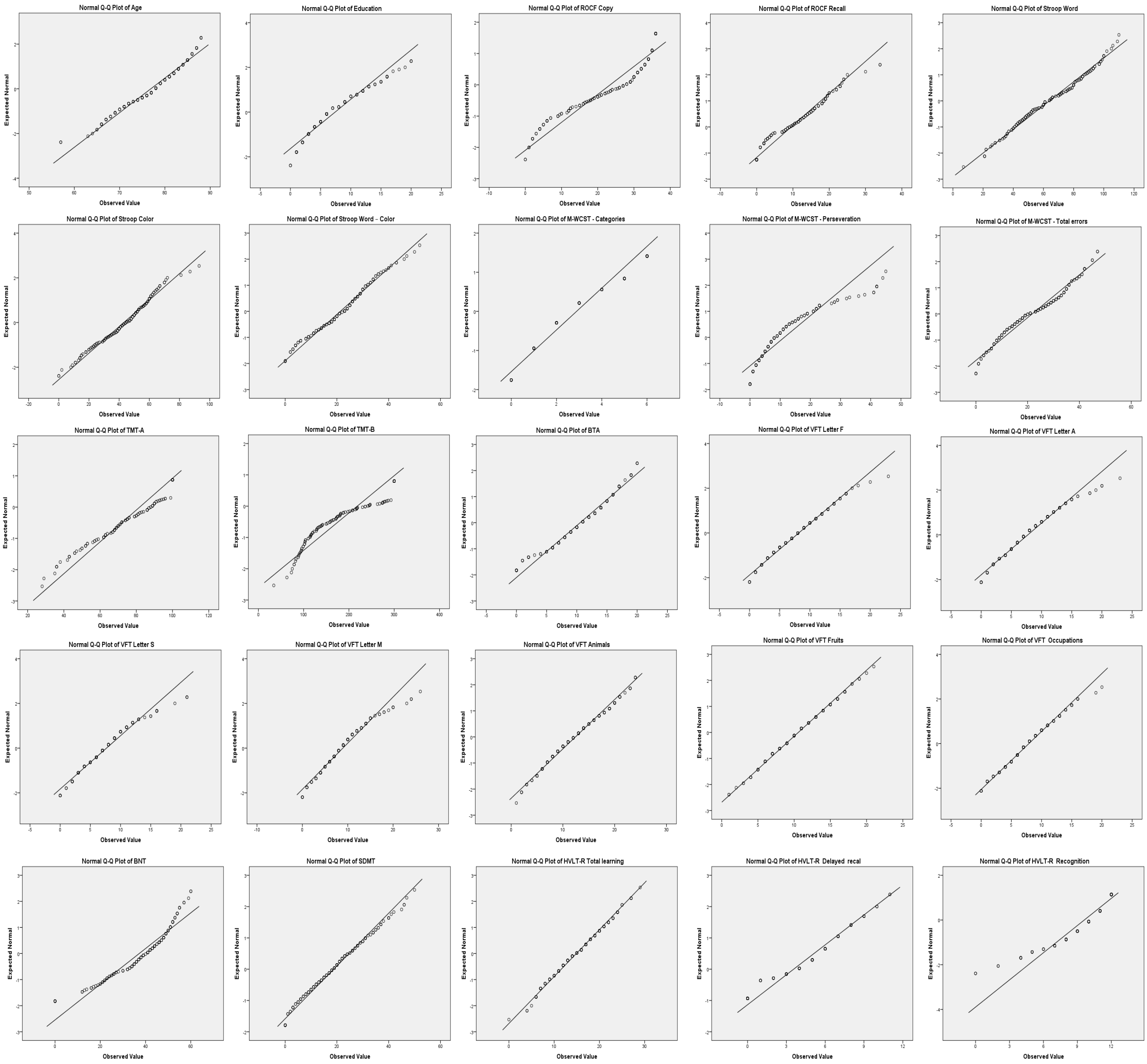

Figure S2. Q-Q plot by each test-score.

Table S2. Normality and homoscedasticity assumptions by each test-score

Scores	Normality		Homoscedasticity	
	Kolmogorov-Smirnov Statistic	Sig.	Levene Statistic	Sig.
Age	0.109	< 0.001	0.076	0.783
Education	0.184	< 0.001	0.007	0.936
ROCF Copy	0.173	< 0.001	24.031	< 0.001
ROCF Recall	0.148	< 0.001	61.133	< 0.001
Stroop Word	0.070	0.036	0.003	0.954
Stroop Color	0.069	0.039	11.274	0.001
Stroop Word – Color	0.071	0.029	1.203	0.274
Stroop Interference	0.054	0.200	8.506	0.004
M-WCST - Categories	0.182	< 0.001	12.558	< 0.001
M-WCST - Perseveration	0.156	< 0.001	0.968	0.326
M-WCST - Total errors	0.080	0.008	7.489	0.007
TMT-A	0.202	< 0.001	19.140	< 0.001
TMT-B	0.255	< 0.001	68.591	< 0.001
BTA	0.084	0.004	28.301	< 0.001
VFT Letter F	0.063	0.090	0.041	0.840
VFT Letter A	0.101	< 0.001	0.008	0.930
VFT Letter S	0.120	< 0.001	0.859	0.355
VFT Letter M	0.111	< 0.001	0.253	0.616
VFT Animals	0.068	0.044	0.563	0.454
VFT Fruits	0.074	0.019	0.740	0.391
VFT Occupations	0.092	0.001	0.136	0.713
BNT	0.118	0.000	4.410	0.037
SDMT	0.057	0.200	7.271	0.008
HVLT-R Total learning	0.066	0.058	0.514	0.474
HVLT-R Delayed recall	0.219	< 0.001	36.070	< 0.001
HVLT-R Recognition	0.188	< 0.001	89.119	< 0.001

Note: ROCF = Rey-Osterrieth Complex Figure; M-WCST = Modified Wisconsin Cart Sorting Test; TMT-A = Trail Making Test- A; TMT-B = Trail Making Test -B; BTA= Brief Test of Attention; VFT = Verbal Fluency Test; BNT= Boston Naming Test; SDMT = Symbol Digit Modalities Test; HVLT-R = Hopkins Verbal Learning Test – Revised.
